# RRAD inhibits the Warburg effect through negative regulation of the NF-κB signaling

**DOI:** 10.18632/oncotarget.3719

**Published:** 2015-03-30

**Authors:** Juan Liu, Cen Zhang, Rui Wu, Meihua Lin, Yingjian Liang, Jia Liu, Xiaolong Wang, Bo Yang, Zhaohui Feng

**Affiliations:** ^1^ Department of Radiation Oncology, Rutgers Cancer Institute of New Jersey, Rutgers, State University of New Jersey, New Brunswick, NJ, USA; ^2^ Institute of Pharmacology and Toxicology, College of Pharmaceutical Sciences, Zhejiang University, Hangzhou, China

**Keywords:** RRAD, the Warburg effect, NF-κB, p65, GLUT1

## Abstract

Cancer cells preferentially use aerobic glycolysis to meet their increased energetic and biosynthetic demands, a phenomenon known as the Warburg effect. Its underlying mechanism is not fully understood. RRAD, a small GTPase, is a potential tumor suppressor in lung cancer. RRAD expression is frequently down-regulated in lung cancer, which is associated with tumor progression and poor prognosis. Recently, RRAD was reported to repress the Warburg effect, indicating that down-regulation of RRAD expression is an important mechanism contributing to the Warburg effect in lung cancer. However, the mechanism by which RRAD inhibits the Warburg effect remains unclear. Here, we found that RRAD negatively regulates the NF-κB signaling to inhibit the GLUT1 translocation and the Warburg effect in lung cancer cells. Mechanically, RRAD directly binds to the p65 subunit of the NF-κB complex and inhibits the nuclear translocation of p65, which in turn negatively regulates the NF-κB signaling to inhibit GLUT1 translocation and the Warburg effect. Blocking the NF-κB signaling largely abolishes the inhibitory effects of RRAD on the translocation of GLUT1 to the plasma membrane and the Warburg effect. Taken together, our results revealed a novel mechanism by which RRAD negatively regulates the Warburg effect in lung cancer cells.

## INTRODUCTION

Majority of cancer cells preferentially use aerobic glycolysis instead of oxidative phosphorylation to meet their increased energetic and biosynthetic demands. This switch from oxidative phosphorylation to aerobic glycolysis is a hallmark of cancer cells, which has long been known as the Warburg effect [1-4]. The Warburg effect has been recently demonstrated to be a key contributor to tumorigenesis, and can be targeted for cancer therapy [3-5]. However, the molecular mechanism underlying the Warburg effect in cancer cells is not fully understood. Recently, many oncogenes or tumor suppressor genes have been reported to be involved in the metabolic switch of cancer cells to aerobic glycolysis. For instance, oncogenes AKT, c-Myc, Ras and HIF-1α promote the Warburg effect, whereas tumor suppressors p53 and PTEN inhibit the Warburg effect in cancer cells [4-8].

RRAD, a member of the Ras-like small GTPase family, was initially identified as a gene associated with Type II diabetes since it was found to be overexpressed in some Type II diabetic patients [9]. RRAD overexpression reduced insulin-stimulated glucose uptake in cultured muscle and adipocytes cells [10]. Recent studies have suggested a tumor suppressive function of RRAD in some types of human cancers. RRAD was found to be frequently down-regulated in different types of human cancers, including lung cancer, breast cancer, and nasopharyngeal carcinoma, etc, due to the hypermethylation of its promoter [11-15]. Furthermore, the down-regulation of RRAD was reported to be associated with tumor progression and poor prognosis in cancer patients [11-15]. Recently, RRAD was identified as p53-regulated gene, which mediates p53′s function in inhibition of cancer metastasis [12]. Our recent study showed that RRAD negatively regulates glycolysis through the inhibition of the translocation of glucose transporter 1 (GLUT1) to the plasma membrane in lung cancer cells [16]. Glucose transporters (GLUTs) mediate the transport of glucose across the plasma membrane of cells, which is the first rate-limiting step for glucose metabolism. GLUT1 is widely expressed in almost all types of cells and tissues, and is responsible for their basal glucose uptake [17]. Ectopic expression of RRAD down-regulates glycolysis whereas knockdown of endogenous RRAD enhances glycolysis [16]. These findings strongly suggest that the down-regulation of RRAD expression could be an important mechanism contributing to the Warburg effect in human lung cancer cells. However, the mechanism by which RRAD inhibits GLUT1 translocation and the Warburg effect remains unclear.

In this study, we demonstrated that RRAD directly binds to the p65 subunit of the NF-κB complex and inhibits p65 nuclear translocation to negatively regulate the NF-κB signaling. Through down-regulating the NF-κB signaling, RRAD inhibits the GLUT1 translocation to the plasma membrane and the Warburg effect in lung cancer cells. Blocking the NF-κB signaling largely abolishes the inhibitory effects of RRAD on GLUT1 translocation to the plasma membrane and therefore the Warburg effect. Thus, our results revealed a novel mechanism by which RRAD negatively regulates the Warburg effect in lung cancer cells.

## RESULTS

### RRAD binds to p65 and inhibits the NF-κB signaling

Our previous study has shown that RRAD negatively regulates the glycolysis through inhibiting GLUT1 translocation to the plasma membrane in human lung cancer cells [16]. However, it remains unclear how RRAD inhibits GLUT1 translocation to negatively regulate glycolysis in cells. RRAD has been reported to exert many functions through its interaction with other proteins in cells [18-20]. To investigate the mechanism by which RRAD represses the glycolysis, we screened for the potential interacting proteins for RRAD by immunoprecipitation (IP) combined with liquid chromatography-tandem mass spectrometry (LC-MS/MS) assays. Human lung cancer H1299-RRAD cells which stably transduced with a pLPCX-RRAD-Flag vector to express RRAD-Flag and their control cells stably transduced with a control pLPCX vector (H1299-con) were employed for assays. Interestingly, LC-MS/MS assays identified p65, a subunit of the transcription factor nuclear factor κB (NF-κB) complex, as a potential binding protein for RRAD.

**Figure 1:**
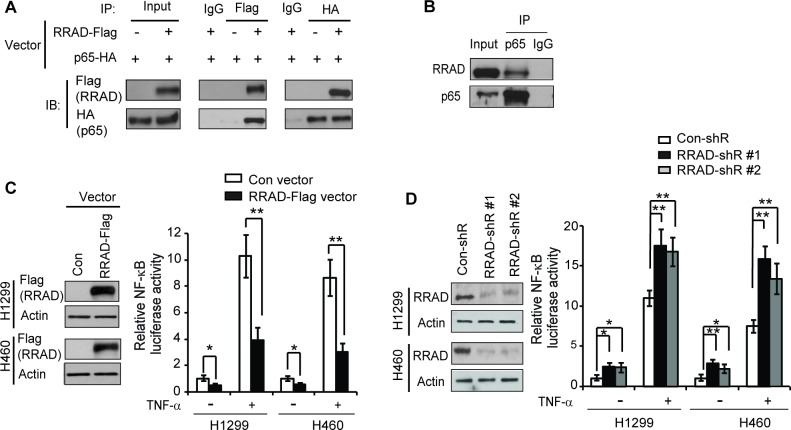
RRAD binds to p65 and inhibits the NF-κB signaling (**A**) Ectopic RRAD-Flag protein interacted with ectopic p65-HA protein in H1299 cells as detected by Co-IP assays. H1299 cells with stable RRAD-Flag overexpression were transfected with p65-HA expression vectors. IP assays were performed with the antibodies against Flag and HA, respectively. (**B**) Endogenous RRAD protein interacted with endogenous p65 protein in H1299 cells as detected by Co-IP assays. (**C**) Ectopic RRAD expression repressed luciferase activities of the NF-κB reporter vectors in H1299 and H460 cells. Left panels: ectopic RRAD-Flag expression detected by Western-blot assays in H1299 and H460 cells stably transduced with pLPCX-RRAD-Flag retroviral vectors (RRAD) or control vectors (Con). (**D**) Knockdown of endogenous RRAD increased luciferase activities of the NF-κB reporter vectors in H1299 and H460 cells. Left panel: RRAD knockdown by stable transduction of 2 different shRNA vectors (RRAD-shR) in H1299 and H460 cells detected by Western-blot assays. Right panels in C and D: Cells with stable RRAD overexpression or knockdown were transfected with the NF-κB luciferease reporter vectors. At 24 h after transfection, cells were treated with or without TNF-α (10 ng/ml) for 6 h before luciferase activities were measured. The luciferase activities in untreated control cells transfected with control vectors or control shRNA (con-shR) were designated as 1. Data are presented as mean ± S.D. (n=3). *p < 0.05; **p < 0.01(student's *t* test).

The NF-κB and its signaling pathway, which is frequently activated in various types of human cancers, play a pivotal role in tumorgenesis [21, 22]. The activated NF-κB signaling has been reported to promote the Warburg effect in cancer cells, although its underlying mechanism is not well-understood [23-26]. A recent study reported that the NF-κB activation promotes the translocation of GLUT1 to the plasma membrane, which could be an important mechanism for NF-κB to promote the Warburg effect in cancer cells [27]. To confirm the interaction between RRAD and p65, we performed Co-immunoprecipitation (Co-IP) assays in H1299 cells. Ectopically expressed RRAD-Flag formed a complex with ectopically expressed p65-HA in H1299 cells transfected with pCMV-RRAD-Flag and pCMV-p65-HA expression vectors (Figure 1A). This interaction was further confirmed by the observation of the protein complex formed between the endogenous RRAD and p65 in H1299 cells (Figure 1B).

The NF-κB signaling pathway plays a critical role in regulating gene expression [21, 22]. To investigate the effect of RRAD on the transcriptional activity of NF-κB, human lung H1299 and H460 cells with ectopic expression of RRAD or knockdown of endogenous RRAD by shRNA vectors were transfected with the pGL4.32 NF-κB luciferase reporter vector which contains NF-κB responsive DNA elements for luciferase reporter assays. Cells were then treated with or without TNF-α, a cytokine that has a well-known activating effect on the NF-κB signaling [21, 22]. Results from luciferase reporter assays showed that RRAD significantly inhibited the NF-κB transcriptional activities; ectopic expression of RRAD reduced luciferase activities of the NF-κB reporter vector in H1299 and H460 cells treated with or without TNF-α (Figure 1C), Consistently, knockdown of RRAD by shRNA vectors significantly induced the NF-κB luciferase activities in above-mentioned cells under both TNF-α untreated or treated conditions (Figure 1D). These results together indicated that RRAD interacts with p65 and inhibits the NF-κB signaling.

### RRAD negatively regulates the Warburg effect through inhibiting the NF-κB signaling

**Figure 2:**
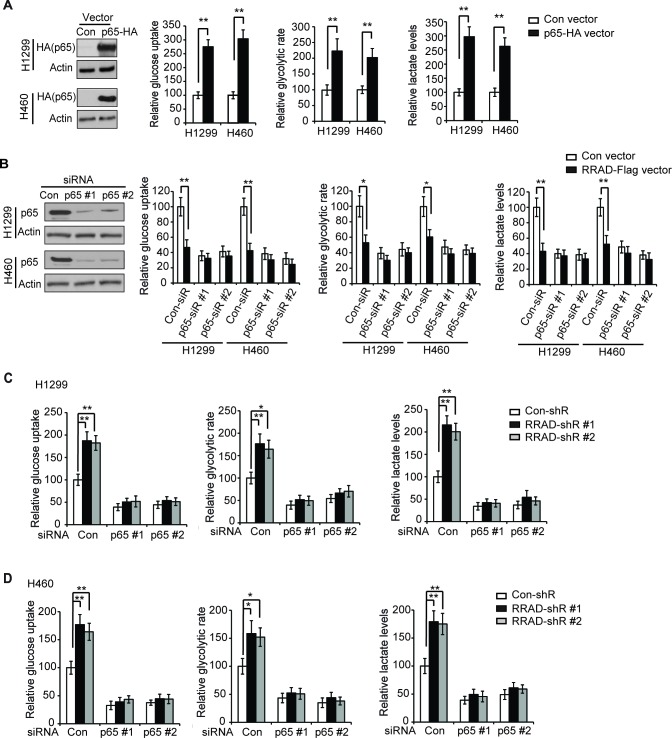
RRAD negatively regulates the Warburg effect through down-regulation of the NF-κB signaling (**A**) Ectopic expression of p65 stimulated glucose uptake, the glycolytic rate and lactate production in H1299 and H460 cells. Cells were transfected with control vectors or pCMV-p65-HA expression vectors for 24 h before assays. Left panel: ectopic p65 expression in H1299 and H460 cells detected by Western-blot assays. (**B**) Knockdown of endogenous p65 largely abolished the inhibitory effect of RRAD on glucose uptake, the glycolytic rate and lactate production in H1299 and H460 cells. Cells with stable ectopic RRAD expression (RRAD) and control cells (Con) were transfected with 2 different siRNA against p65 (p65-siR). Left panel: Western-blot analysis of knockdown of p65 by siRNA in cells. (C, D) p65 knockdown largely abolished the promoting effects of RRAD knockdown on glucose uptake, the glycolytic rate and lactate production in H1299 (**C**) and H460 (**D**) cells. Cells stably transduced with control (con-shR) or RRAD shRNA (RRAD-shR) vectors were transfected with siRNA oligos against p65 (p65-siR) or control siRNA oligos (Con-siR) for 48 h before assays. Data are presented as mean ± S.D. (n=3). *p < 0.05; **p < 0.01(student's *t* test).

The NF-κB activation has been reported to promote the Warburg effect in cancer cells [23-26]. Consistent with previous reports [23-26], activation of NF-κB by ectopic expression of p65 with the pCMV-p65-HA vector clearly promoted glucose uptake, the glycolytic rate and lactate production in both H1299 and H460 cells (Figure 2A). Here, we investigated whether RRAD down-regulates the Warburg effect through its down-regulation of the NF-κB signaling in lung cancer cells. While ectopic expression of RRAD significantly inhibited the Warburg effect, blocking the NF-κB signaling by knockdown of the endogenous p65 with siRNA oligos largely abolished the inhibitory effects of RRAD overexpression on the Warburg effect in H1299 and H460 cells (Figure 2B). Furthermore, while knockdown of endogenous RRAD by shRNA vectors significantly promoted the Warburg effect in H1299 and H460 cells, knockdown of the endogenous p65 by siRNA oligos largely abolished the promoting effects of RRAD knockdown on the Warburg effect in H1299 (Figure 2C) and H460 cells (Figure 2D). These results strongly suggest that RRAD negatively regulates the Warburg effect through its down-regulation of the NF-κB signaling.

### RRAD inhibits GLUT1 translocation through inhibiting the NF-κB signaling

**Figure 3:**
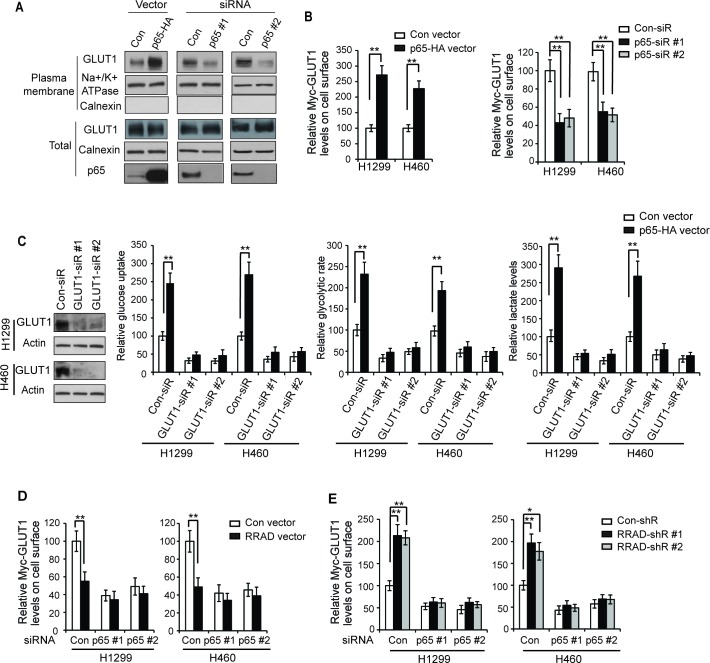
RRAD inhibits GLUT1 translocation to the plasma membrane through down-regulating NF-κB signaling (**A**) The NF-κB signaling promoted the translocation of endogenous GLUT1 to the plasma membrane in H1299 cells detected by Western-blot assays. Cells were transfected with pCMV-p65-HA expression vectors (left panel) or p65 siRNA oligos to knock down p65 (right panel), and the endogenous GLUT1 levels in the isolated plasma membrane fraction and whole cell lysates were analyzed by Western-blot assays. (**B**) Ectopic expression of p65 promoted the translocation of Myc-GLUT1 to the plasma membrane (left panel), whereas p65 knockdown inhibited the translocation of Myc-GLUT1 to the plasma membrane (right panel) in H1299 and H460 cells. Cells were transfected with p65 expression vectors or p65-siR together with Myc-GLUT1 vectors. The levels of Myc-GLUT1 on cell surface were analyzed in a flow cytometer and normalized with the total Myc-GLUT1 levels in cells. (**C**) GLUT1 knockdown greatly abolished the promoting effects of p65 on glucose uptake, the glycolytic rate and lactate production in H1299 and H460 cells. Cells were pre-transfected with 2 different GLUT1 siRNAs (GLUT1-siR) before transfection with p65 expression vectors. Left panel: Western-blot analysis of knockdown of GLUT1 by siRNA in cells. (**D**) p65 knockdown largely abolished the inhibitory effects of RRAD overexpression on Myc-GLUT1 translocation to cell surface in cells measured by flow cytometry. H1299 and H460 cells with stable RRAD overexpression (RRAD) and control cells (Con) were pre-transfected with p65-siR before transfection of Myc-GLUT1 vectors. (**E**) p65 knockdown largely abolished the promoting effects of RRAD knockdown on Myc-GLUT1 translocation to cell surface. H1299 and H460 cells stably transduced with 2 different RRAD shRNA vectors (RRAD-shR) and control shRNA (Con-shR) were pre-transfected with p65-siR before transfection of Myc-GLUT1 vectors. Data are presented as mean value ± SD (n=3). *p < 0.05; **p < 0.01 (student's *t* test).

A recent study reported that the activated NF-κB signaling promotes the translocation of GLUT1 to the plasma membrane to facilitate glucose uptake, which could be an important mechanism by which NF-κB activates the Warburg effect [27]. Together with our finding that RRAD negatively regulates the NF-κB signaling, these findings raised a possibility that RRAD inhibits the GLUT1 translocation to the plasma membrane through its negative regulation of the NF-κB signaling, which could be an important mechanism by which RRAD inhibits GLUT1 translocation. Consistent with this previous report [27], our results clearly showed that the NF-B activation promoted the GLUT1 translocation to the plasma membrane and therefore enhanced the Warburg effect in lung cancer cells. Ectopic expression of p65 by the pCMV-p65 expression vector greatly promoted the translocation of endogenous GLUT1 to the plasma membrane as shown by Western-blot assays using the isolated plasma membrane fraction of H1299 and H460 cells (Figure 3A). Furthermore, knockdown of p65 by siRNA reduced the translocation of endogenous GLUT1 to the plasma membrane in cells (Figure 3B). To confirm this result, cells with p65 overexpression or knockdown were transduced with pLPCX-Myc-GLUT1 vectors that express GLUT1 with Myc tag in its first exofacial loop, and the levels of Myc-GLUT1 on the cell surface or in the whole cell were measured by immunofluorescence (IF) staining with an anti-Myc antibody followed by flow cytometry analysis. While p65 overexpression or knockdown did not affect the total levels of Myc-GLUT1 in cells, p65 overexpression significantly increased the levels of Myc-GLUT1 protein on the cell surface (Figure 3B, left panel), whereas p65 knockdown significantly reduced the levels of Myc-GLUT1 protein on the cell surface (Figure 3B, right panel). Our results further showed that knockdown of the endogenous GLUT1 by siRNA largely abolished the promoting effects of NF-κB activation on glucose uptake, the glycolytic rate and lactate production in H1299 and H460 cells with overexpression of p65 (Figure 3C). These results together indicate that activated NF-κB signaling promotes the translocation of GLUT1 to the plasma membrane to facilitate glucose uptake, which is an important mechanism for NF-κB to activate the Warburg effect in cancer cells.

We further investigated whether RRAD inhibits GLUT1 translocation through negative regulation of the NF-κB signaling. As shown in Figure 3D and 3E, blocking the NF-κB signaling by knockdown of endogenous p65 largely abolished the inhibitory effects of RRAD overexpression on GLUT1 translocation in H1299 and H460 cells (Figure 3D), and also largely abolished the promoting effects of RRAD knockdown on GLUT1 translocation (Figure 3E). These results strongly suggest that RRAD inhibits GLUT1 translocation to the plasma membrane mainly through its inhibition of the NF-κB signaling, which is an important mechanism for RRAD to inhibit the Warburg effect.

### RRAD interacts with p65 through its N-terminus

**Figure 4:**
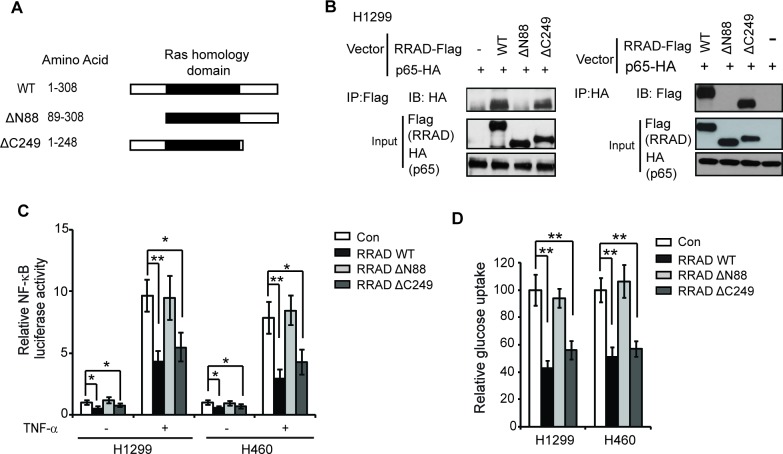
RRAD interacts with p65 through the N-terminus (**A**) Schematic representation of RRAD deletion mutants. pCMV-Flag vectors expressing WT RRAD or two deletion mutants were constructed. (**B**) RRAD-ΔC249-Flag but not RRAD-ΔN88-Flag interacted with p65-HA. H1299 cells were transfected with WT or mutant RRAD-Flag vectors together with p65-HA vectors followed by Co-IP. Co-IP assays were performed with the antibodies against Flag (Left panel) or HA (Right panel). (**C**) Ectopic expression of RRAD-ΔC249 but not RRAD-ΔN88 inhibited the transcriptional activity of NF-κB. Cells transfected with indicated vectors together with the NF-κB luciferase reporter vectors. At 24 h after transfection, cells were treated with or without TNF-α (10 ng/ml) for 6 h before luciferase activities were measured. (**D**) Ectopic expression of RRAD-ΔC249 but not RRAD-ΔN88 inhibited glucose uptake in H1299 and H460 cells. Cells were transfected with indicated vectors for 24 h before glucose uptake assays. Data are presented as mean ± S.D. (n=3). *p < 0.05; **p < 0.01 (student's *t* test).

To define the binding region of RRAD with p65, two Flag-tagged deletion mutants of RRAD were constructed and cloned into pCMV-Flag vectors, including ΔN88 (deletion of the N-terminal 88 amino acids) and ΔC249 (deletion of the C-terminal 59 amino acids) (Figure 4A). Both N-terminus and C-terminus of RRAD have been reported to be important for interacting with other proteins [18-20]. These two mutant vectors or wild-type (WT) RRAD-Flag expression vectors were transfected into H1299 cells together with the pCMV-p65-HA vector, and their binding to p65-HA protein were analyzed by Co-IP assays. As shown in Figure 4B, deletion of C-terminal residues 249-308 (ΔC249 mutant) did not significantly affect the ability of RRAD-Flag to bind to p65-HA. In contrast, deletion of N-terminal residues (ΔN88 mutant) largely abolished the RRAD binding to p65-HA. Furthermore, deletion of C-terminal residues 249-308 (ΔC249 mutant) did not significantly reduce the inhibitory effects of RRAD on the luciferase activities of the NF-κB (Figure 4C) and glucose uptake (Figure 4D) in H1299 and H460 cells. In contrast, deletion of N-terminal residues (ΔN88 mutant) largely abolished the inhibitory effects of RRAD on luciferase activities of the NF-κB (Figure 4C) and glucose uptake (Figure 4D) in cells. These results indicate that the N-terminus of RRAD is essential for the interaction of RRAD with p65, as well as for the function of RRAD in inhibiting GLUT1 translocation and the Warburg effect.

### RRAD inhibits TNF-α-induced nuclear translocation of the p65

**Figure 5:**
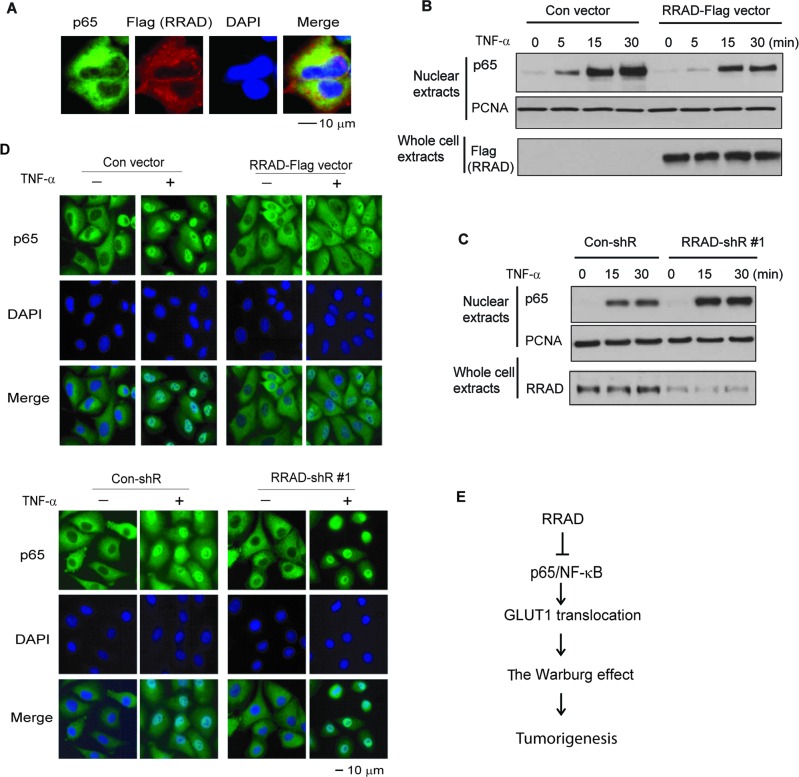
RRAD inhibits the nuclear translocation of p65 (**A**) Immunofluorescence images of colocalization of endogenous p65 and ectopic RRAD-Flag in the cytoplasm of H1299 cells. H1299 cells stably transduced with the pLPCX-RRAD-Flag vector were used for IF staining. (**B**) Ectopic expression of RRAD inhibited the p65 nuclear translocation induced by TNF-α in H1299 cells measured by Western-blot assays. H1299 cells with stable ectopic RRAD expression (RRAD) and control cells (Con) were treated with TNF-α (10 ng/ml) and collected at different time points after treatment. (**C**) Knockdown of endogenous RRAD promoted the p65 nuclear translocation induced by TNF-α. H1299 cells stably transduced with shRNA vectors against RRAD (RRAD-shR) or control shRNA (Con-shR) were treated with TNF-α (10 ng/ml) and collected at different time points after treatment. (**D**) RRAD overexpression inhibited p65 nuclear translocation (upper panels), whereas RRAD knockdown promoted p65 nuclear translocation (lower panels) in H1299 cells detected by IF staining. Cells were treated with TNF-α (10 ng/ml) for 15 min before IF staining. Nuclei were stained with DAPI. In C and D, two different RRAD-shR were used and very similar results were observed. For the sake of clarity, results from one RRAD-shR are presented. (**E**) Schematic depicting that RRAD negatively regulates the Warburg effect through inhibiting the NF-κB signaling.

The NF-κB is normally kept in an inactive state in the cytoplasm. In response to stimuli, such as TNF-α treatment, NF-κB is translocated to the nucleus, where it activates gene transcription [21, 22]. IF staining results showed that RRAD-Flag was mainly localized in the cytoplasm, where it colocalized with p65 in H1299 cells untreated with TNF-α (Figure 5A). While TNF-α greatly promoted the p65 nuclear translocation in H1299 cells, ectopic expression of RRAD greatly reduced the p65 nuclear translocation induced by TNF-α in H1299 cells as detected by Western-blot assays (Figure 5B). Furthermore, knockdown of endogenous RRAD by shRNA vectors clearly promoted the TNF-α-induced p65 nuclear translocation (Figure 5C). IF staining assays showed the consistent results in H1299 cells (Figure 5D). Taken together, these results strongly suggest that RRAD interacts with p65 and inhibits its nuclear translocation to negatively regulate NF-κB activities, which in turn represses the Warburg effect in cancer cells (Figure 5E).

## DISSCUSSION

The Warburg effect is one of the hallmarks of cancer cells [1, 2, 6]. The switch from oxidative phosphorylation to glycolysis provides a critical mechanism for cancer cells to meet their dramatically increased energetic and biosynthetic demands to support their rapid growth and proliferation. Understanding the mechanism underlying the Warburg effect could lead to the development of new strategies for cancer therapy. The evidence that many oncogenes or tumor suppressor genes are involved in the metabolic switch of cancer cells to glycolysis indicates that genetic alterations during tumorigenesis are also playing critical roles in regulation of the Warburg effect in cancer cells [5, 6]. The NF-κB signaling pathway is frequently activated in various types of human cancers, including lung cancer, and plays an important role in tumor initiation and progression [21, 22]. The activated NF-κB signaling has been reported to promote the Warburg effect in cancer cells, although its underlying mechanism is not well-understood [23]. Recently, the NF-κB activation was reported to promote the translocation of GLUT1 to the plasma membrane, which could be an important mechanism for NF-κB to promote the Warburg effect in cancer cells [27].

RRAD was recently reported to play a potential role in tumor suppression, particularly in lung cancer [11-14]. Its expression is frequently down-regulated in different types of cancers, including lung cancer, and the hypermethylation of its promoter is an important mechanism [11-14]. Restoration of RRAD expression in cancer cells inhibits tumor growth and metastasis [11-14]. Our previous study showed that RRAD inhibits glycolysis through inhibiting GLUT1 membrane translocation in lung cancer cells, which suggests that the frequently-observed down-regulation of RRAD expression in lung cancer could be an important mechanism for the Warburg effect in lung cancer cells. However, its underlying mechanism was unclear.

In this study, we investigated the mechanism by which RRAD inhibits GLUT1 translocation and the Warburg effect in lung cancer cells. Using LC-MS/MS assays, we screened for the potential interacting proteins for RRAD, which led to the identification of p65 as an important binding protein of RRAD. This interaction resulted in the inhibition of p65 nuclear localization and therefore the reduced transcription activities of NF-κB. Our results confirmed that the activation of NF-κB promoted GLUT1 translocation to the plasma membrane as an important mechanism by which NF-κB activates the Warburg effect in cancer cells. Furthermore, we found that RRAD inhibited the GLUT1 translocation and the Warburg effect mainly through the down-regulation of NF-κB activities. Blocking the NF-κB signaling largely abolished the inhibitory effects of RRAD on GLUT1 translocation and the Warburg effect. Currently, it still remains largely unclear how NF-κB regulates GLUT1 translocation. Future studies should shed further light on its detailed molecular mechanism. In summary, the results from this study demonstrated that RRAD inhibits GLUT1 translocation and glycolysis through its direct interaction with p65 and inhibition of the NF-κB signaling, which revealed a novel mechanism by which RRAD negatively regulates the Warburg effect in cancer.

## MATERIALS AND METHODS

### Cells and vectors

Human lung epithelial cancer H1299 and H460 cells were purchased from ATCC (Manassas, VA). For cells with stable ectopic RRAD overexpression, cells were transduced with a pLPCX-RRAD-Flag retroviral vector and selected by puromycin [16]. The pCMV-RRAD-Flag WT and deletion mutations were constructed by PCR amplification. pCMV-p65-HA vector was constructed by using DNA fragment from pCMV4-p65 (Addgene). The lentiviral shRNA vectors against human RRAD (V3LHS_364015 and V3LHS_409093) were obtained from Open Biosystems (Huntsville, AL). To avoid off-target effects, two different siRNA oligos against each gene were employed for all knockdown experiments. The siRNA oligos against human GLUT1 (5′- CGAACTATGAACTACAAAGCTTCTA-3′ and 5′-TCAAAGTTCCTGAGACTAAA GGCCG-3′) and human p65 (5′-GGAGTACCCTGAGGCTATAACTCGC-3′ and 5′- AGCACAGATACCACCAAGACCCACC-3′) were obtained from Integrated DNA Technologies. Vectors and siRNA oligos were transfected into cells using Lipofectamine 2000 (Invitrogen).

### LC-MS/MS analysis

To determine the potential RRAD binding proteins, RRAD-Flag protein in H1299-RRAD cells was pulled down by IP using anti-Flag (M2) beads and eluted with 0.1M glycine solution. H1299-con cells were used as a control. Eluted materials were separated in a 4-16% SDS-PAGE gel and visualized by silver staining using the silver staining kit (Invitrogen). LC-MS/MS analysis was performed at the Biological Mass Spectrometry facility of Rutgers University.

### Co-immunoprecipitation (Co-IP) assays

Co-IP assays were performed as we previously described [28]. In brief, cell lysates were prepared using NP-40 buffer (50mM Tris-HCl pH 7.5, 1% NP-40, 150mM NaCl, 5 mM EDTA) and incubated with IP matrix (Santa Cruz) conjugated to IP antibody (anti-HA, Santa Cruz; or anti-Flag M2, Sigma) for overnight. The IP matrix-antibody complex was then washed with NP-40 buffer, and protein complexes were eluted and subjected to Western-blot assays.

### Western-blot assays

Western-blot assays were performed as we previously described [16, 29]. Following antibodies were employed for assays: anti-RRAD (a generous gift from Dr. CR Kahn, Harvard Medical School); anti-Flag (Sigma); anti-HA (Santa Cruz); anti-p65 (Santa Cruz); anti-GLUT1 (Abcam); anti- Na^+^/K^+^ ATPase (Novus); anti-PCNA (Santa Cruz); anti-Calnexin (Abcam), and anti-Actin (Sigma). Western-blot results were analyzed by using Image J 1.45s software (NIH).

### NF-κB luciferase reporter assays

For luciferase activity assays, the NF-κB luciferase reporter vector, pGL4.32 (luc2P/NF-κB-RE/Hygro, Promega), which contains five copies of an NF-κB responsive DNA element that drives transcription of the luciferase reporter gene was employed. Cells were co-transfected with the luciferase reporter vector and pRL-null vector expressing renilla luciferase as an internal control to normalize the transfection efficiency using Lipofectamine 2000 (Invitrogen). At 24 h after transfection, cells were treated with or without TNF- (10 ng/ml) for 6 h before luciferase assays. The luciferase activities were detected as previously described [30, 31].

### Measurement of glucose uptake, the glycolytic rate and lactate production in cells

Glucose uptake in cells was analyzed by measuring the uptake of ^3^H-2-deoxyglucose [30, 32]. Briefly, cells cultured in 12-well plates were pre-incubated in glucose-free media for 30 min before ^3^H-2-deoxyglucose (1 μCi/well) was added to the cells. After incubation for 30 min, cells were washed with PBS and lysed in 1% SDS. The radioactivity of cell lysates was determined in a liquid scintillation counter and normalized to the protein concentrations of cell lysates. The glycolytic rate in cells was measured by monitoring the conversion of 5-^3^H-glucose to ^3^H_2_O [30, 33]. Briefly, cells (1×10^6^) were collected and washed in PBS before they were resuspended in 1 mL of Krebs buffer without glucose for 30 min. Cells were collected and resuspended in 0.5 mL of Krebs buffer containing 10 mM glucose and 5 μCi of 5-[^3^H]glucose for 1 h. Triplicate 100 μL aliquots were transferred to uncapped PCR tubes containing 100 μL of 0.2 N HCl, and a tube was transferred to a scintillation vial containing 0.5 mL of H_2_O. The scintillation vials were sealed and left for 48 h. The amounts of diffused and undiffused ^3^H were then determined in a liquid scintillation counter. The cell lactate production levels were determined by using a Lactate Assay Kit (Biovision) [30]. In brief, cells were cultured in fresh phenol red-free media and incubated for 24 h. The lactate levels in culture media were determined by using lactate Assay Kits, and normalized with cell number.

### Analysis of endogenous levels of GLUT1 on plasma membrane

The plasma membrane fraction of cells was isolated from the other membrane fractions of cells which include the endoplasmic reticulum (ER) as we previously described [16, 34]. The levels of GLUT1 in the plasma membrane fraction were measured by Western-blot assays. Na^+^/K^+^ ATPase, a plasma membrane protein, was detected as an internal standard. Calnexin, an ER membrane protein, was detected to exclude the contamination of plasma membrane by the other membrane fractions that include the ER. Whole cell extracts were used to measure the total GLUT1 levels in cells.

### Analysis of the levels of Myc-GLUT1 on the plasma membrane

Cells were transduced with pLPCX-Myc-GLUT1 vectors which express the GLUT1 with Myc tag in the first exofacial loop [16, 34]. At 48 h after transduction, the levels of Myc-GLUT1 on the cell surface and in whole cells were measured by IF staining in a flow cytometer as we previously described [16, 34]. The relative levels of Myc-GLUT1 on the cell surface were calculated after normalization with the total levels of Myc-GLUT1 in cells. Cells transduced with empty pLPCX vectors were used as negative controls.

### Analysis of p65 nuclear translocation

H1299 cells were treated with TNF-α (10 ng/ml) and collected at different time points. The nuclei were isolated by employing CelLytic NuCLEAR Extraction kit (Sigma) according to the manufacturer's instructions. Western-blot assays and anti-p65 antibody (Cell Signaling Technology) was employed to measure the levels of p65 protein in nuclear extracts. Nuclear protein PCNA was detected by PCNA antibody (Santa Cruz) as an internal standard.

### Statistical analysis

All *P* values were obtained using a student *t*-test. *: *p* < 0.05; **: *p* < 0.01.
